# Charge Injection, Carriers Recombination and HOMO Energy Level Relationship in Perovskite Solar Cells

**DOI:** 10.1038/s41598-017-06245-5

**Published:** 2017-07-21

**Authors:** Jesús Jiménez-López, Werther Cambarau, Lydia Cabau, Emilio Palomares

**Affiliations:** 1 Institute of Chemical Research of Catalonia (ICIQ), Barcelona Institute of Science and Technology, Avda. Països Catalans, 16, E-43007 Tarragona, Spain; 2 ICREA, Passeig Lluis Companys, 28, E-08018 Barcelona, Spain

## Abstract

We present a comparative study between a series of well-known semiconductor polymers, used in efficient organic solar cells as hole transport materials (HTM), and the state-of-the art material used as hole transport material in perovskite solar cells: the spiro-OMeTAD. The observed differences in solar cell efficiencies are studied in depth using advanced photoinduced spectroscopic techniques under working illumination conditions. We have observed that there is no correlation between the highest occupied molecular orbital (HOMO) energy levels of the organic semiconductors and the measured open-circuit voltage (V_OC_). For instance, spiro-OMeTAD and P3HT have a comparable HOMO level of ~5.2 eV vs vacuum even though a difference in V_OC_ of around 200 mV is recorded. This difference is in good agreement with the shift observed for the charge vs voltage measurements. Moreover, hole transfer from the perovskite to the HTM, estimated qualitatively from fluorescence quenching and emission lifetime, seems less efficient for the polymeric HTMs. Finally, the recombination currents from all devices were estimated by using the measured charge (calculated using photoinduced differential charging) and the carriers’ lifetime and their value resulted in accordance with the registered short-circuit currents (J_SC_) at 1 sun.

## Introduction

In the last few years, the field of photovoltaic has been flooded by the appearance of perovskite^1^, a wide-ranging class of materials for solar cells which have nowadays pushed its power efficiency beyond 20%^2, 3^ with a certified record of 22.1% under standard measurement conditions^4^. The perovskite materials can be described by the general formula ABX_3_, where A is the organic cation (often methylammonium, CH_3_NH_3_^+^ or formamidinium, CH(NH_2_)_2_)^+^, B is the inorganic cation (typically Pb^2+^), and X is the halide (I^−^, Cl^−^ or Br^−^). A fine-tuning of the perovskite materials and the band-gap has led to the current record efficiency.

Starting from precursor solutions^5–8^ or via multi-source thermal evaporation^9, 10^, various methods have been developed to form a high quality layer of perovskite and many of them actually give rise to stable, reproducible and efficient solar cells also depending on the architecture of the whole device. The most employed configuration of perovskite solar cells (PSC), namely ‘regular’ structure consists of: a transparent conductive fluorine doped tin oxide (FTO); a metal oxide layer which, in turn, can be mesoporous (mesoscopic architecture) or compact (planar architecture) acting as the electron transporting material (ETM); the perovskite absorber; the HTM and a metal contact (usually gold or silver). In addition, PSC have been successfully fabricated also in the ‘inverted’ planar configuration (a typical structure in organic solar cells, OSC), with indium doped tin oxide (ITO) as the transparent conductive electrode.

Since the breakthrough of PSC, a myriad of new organic semiconductor materials have been synthesized and tested as HTM^11–18^. However, so far, none of them have display efficiencies above 20%. In fact, semiconductor polymers, that have provided a step forward towards OSC highest efficiencies, have not achieved performances close to those recorded for spiro-OMeTAD^19^. Hence, the solid state organic semiconductor used as selective contact for holes has remained the well-known spiro-OMeTAD, with the exception of poly(triarylamine) (PTAA)^20, 21^, to achieve top efficiencies.

**Figure 1:**
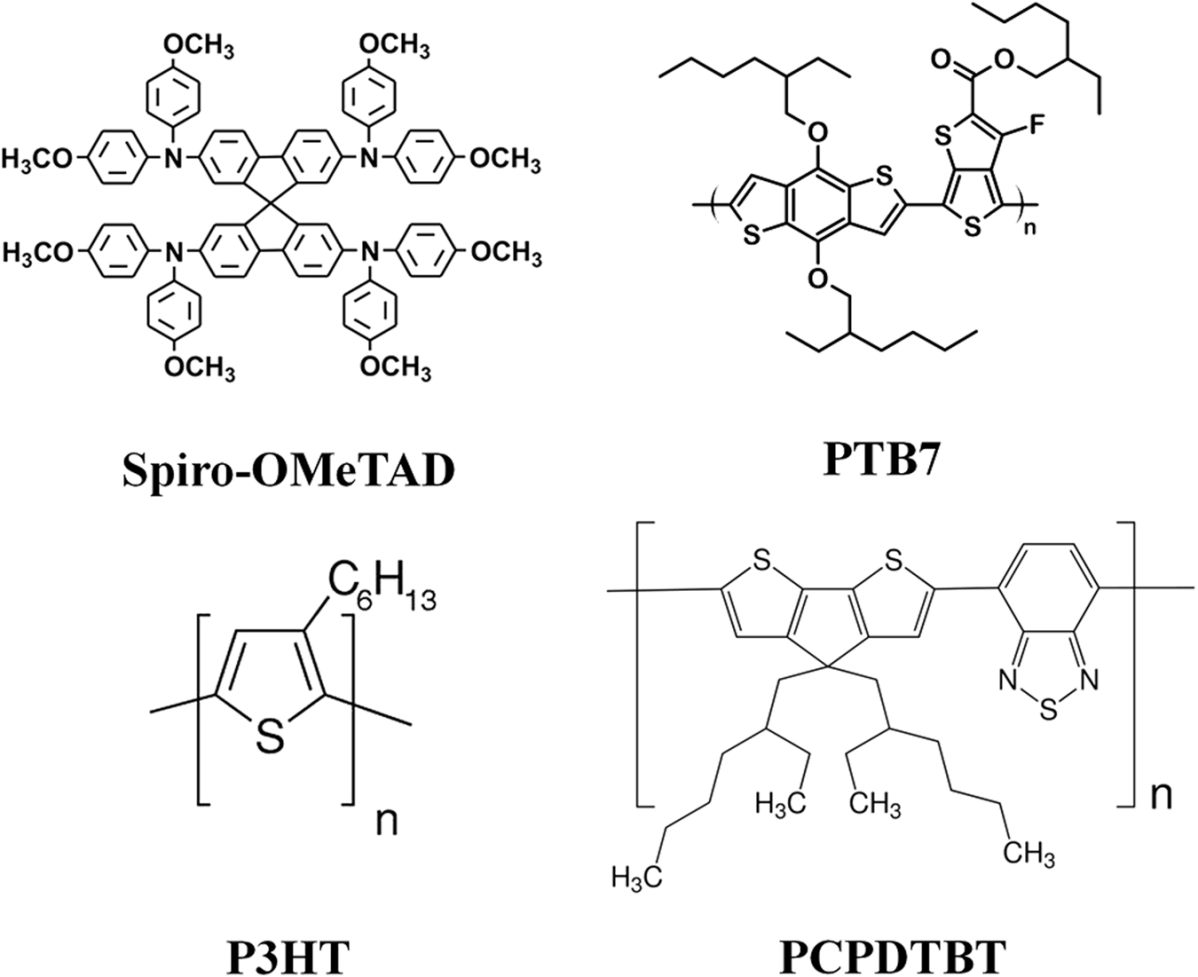
Molecular structure of the HTMs employed in this study.

In this paper we aim to study efficient PSC using three of the most representative semiconductor polymers like poly(3-hexylthiophene-2,5-diyl) (P3HT), poly[2,6-(4,4-bis-(2-ethylhexyl)-4H-cyclopenta[2,1-b;3,4-b′]dithiophene)-alt-4,7(2,1,3-benzothiadiazole)] (PCPDTBT) and poly[[4,8-bis[(2-ethylhexyl)oxy]benzo[1,2-b:4,5-b’]dithiophene-2,6-diyl][3-fluoro-2-[(2-ethylhexyl)carbonyl]thieno[3,4-b]thiophenediyl]] (PTB7), depicted in Fig. 1, and understand the reasons that hamper these materials to reach higher efficiencies when used as hole selective contacts.

Efficiency losses in solar cells account for the presence of radiative recombination within the semiconductor material and interfacial carrier recombination (non-radiative) processes^22–25^. We have measured the charge in the solar cell at different light intensities, which lead to different solar cell V_OC_ (so called light bias). As we demonstrated before^26^, unlike OSC and dye-sensitized solar cells (DSSC), the measured charge in PSC depends on the acquisition method. Photoinduced charge extraction (PICE) is a direct measurement which allows the estimation of the overall charge generated upon illumination while photoinduced differential charging (PIDC) calculates the amount of charge from the so called “differential capacitance”, obtained as a result of the combination of small perturbation transient techniques. The carrier recombination lifetimes have been measured by photoinduced transient photovoltage (PI-TPV) under solar cell open-circuit conditions.

## Results

### Solar cells characterization

**Figure 2:**
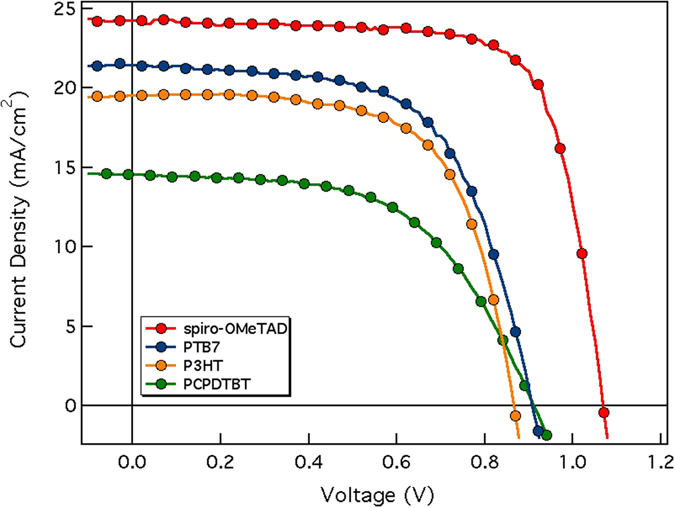
J-V graphs of the devices measured under standard (AM 1.5G) 1 sun conditions from V_OC_ to J_SC_ (champion cells).

The current density-voltage (J-V) characteristics of the best devices are shown in Fig. 2. These measurements were carried out under 1 sun illumination conditions (AM1.5G, 100 mW/cm^2^) and performing the voltage scan in the direction V_OC_ to J_SC_ (reverse sweep). All cells present substantial hysteresis in the J-V (see Table 1 and Figure S1) leading to a difference in photovoltaic parameters depending on the scan direction and rate, as observed by many research groups^27, 28^. The cause for this behavior is yet to be unequivocally determined. Mobile ions migration in the perovskite material has been proposed as a possible explanation for hysteresis by Unger *et al*.^29^, although very recently Belisle at al. demonstrated that this occurrence alone cannot explain the observed phenomenon^30^. In any case, the origin of the hysteresis is not the focus of this work and will not be discussed in this manuscript.

**Table 1:** Photovoltaic parameters of the devices characterized in this study under standard 1 sun illumination.

HTM	Sweep	V_OC_ (mV)	J_SC_ (mA/cm^2^)	FF (%)	PCE (%)
Spiro-OMeTAD	F	936	24.4	55.1	12.6 (9.0 ± 1.5)
	R	1068	24.3	73.6	19.1 (14.9 ± 2.0)
PTB7	F	856	21.3	44.7	8.2 (7.2 ± 1.2)
	R	908	21.4	61.9	12.0 (10.8 ± 1.2)
P3HT	F	807	17.8	34.0	4.9 (4.8 ± 0.8)
	R	866	19.6	65.1	11.0 (10.2 ± 0.9)
PCPDTBT	F	853	13.4	42.8	4.9 (4.6 ± 0.6)
	R	911	14.6	56.0	7.4 (6.5 ± 1.0)

Each value corresponds to the champion cell. The PCE value between parentheses is the average of eight solar cells.

In our case, both V_OC_ and J_SC_ of spiro-OMeTAD cells are greater than the other polymers cells as shown in Fig. 2 (“champion cells”) and reported in Table 1.

It is worthy to mention that spiro-OMeTAD based cells efficiencies are in agreement with the ones reported by most of the groups working on methylammonium lead iodide (MAPI, perovskite bandgap 1.6 eV) solar cells. Regarding polymer based cells, in this study we have surpassed the reported power conversion efficiency (PCE) record for PCPDTBT acting as HTM^20^, while for P3HT and PTB7, the efficiencies obtained are similar to the best efficiencies reported avoiding the use of dopants^31, 32^.

**Figure 3:**
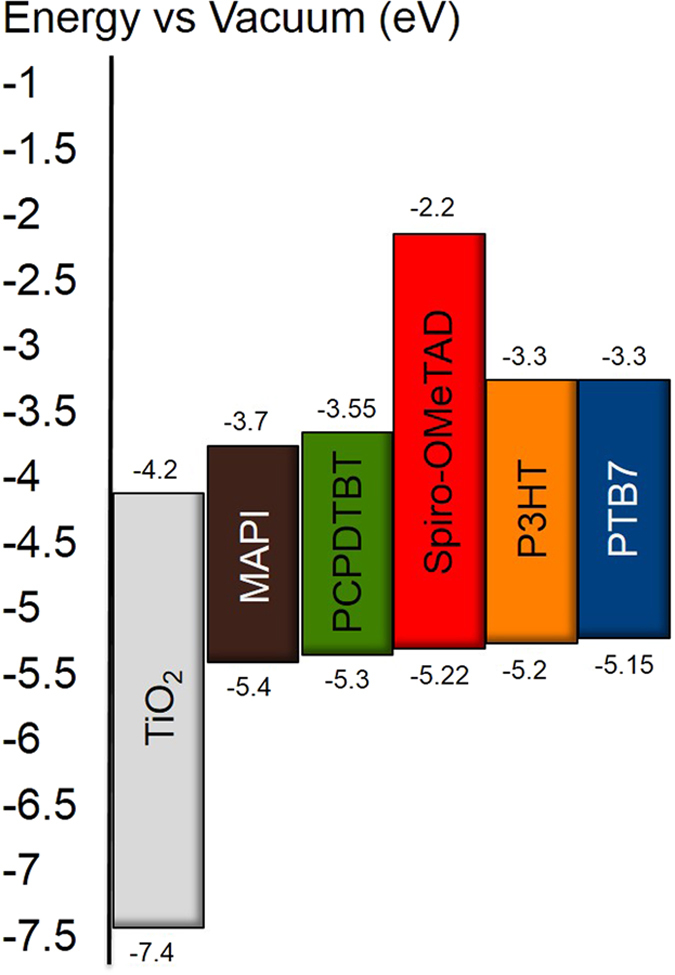
Energy level diagram of the different materials employed.

In Fig. 3 we illustrated the HOMO and lowest unoccupied molecular orbital (LUMO) levels for all the HTM employed in this study as reported by Wright *et al*.^33^. From the values obtained, it appears evident that the differences in V_OC_ of the cells could not be simply related to the differences in the HOMO energy level of the HTM since they are alike.

**Figure 4:**
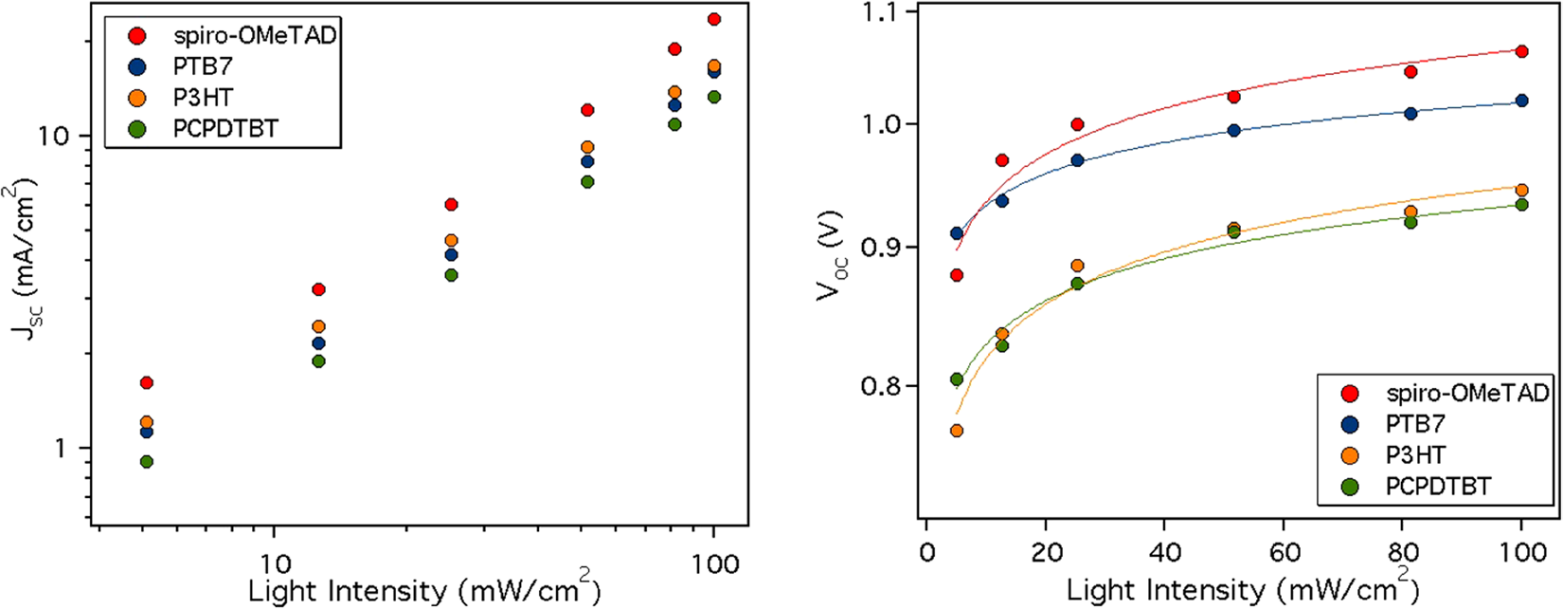
Light intensity dependence of J_SC_ (left) and V_OC_ (right) for the structure FTO/TiO_2_/MAPI/HTM/Au.

We first measured the J_SC_ dependence with light intensity (LI). Figure 4 (left) shows a power law dependence of J_SC_ on LI (J_SC_ proportional to LI^α^ with α close to 1 for each HTM (α_spiro-OMeTAD_ = 0.98; α_PTB7_ = 0.97; α_P3HT_ = 0.92; α_PCPDTBT_ = 0.94). These values indicate minimal light intensity dependence for charge collection, suggesting that free-carriers recombination processes at short-circuit are negligible^34^.

From Fig. 4 (right) we can observe the variation of V_OC_ with LI. The values obtained with the reverse sweeps have been taken into account since they coincide with the V_OC_ obtained after a short stabilization time (see Figure S3). A first approach to the origin of the charge recombination that occurs in a device can be studied by examining the ideality factor (n_id_), which can be obtained from Eq. 1, applied to the dependence of V_OC_ with LI:

1 $${n}_{id}=\frac{e}{kT}\frac{d{V}_{OC}}{dln(LI)}$$

where *e* is the elementary charge and *k*T is the thermal energy. Generally, the ideality factor ranges from 1 to 2, where n_id_ = 1 indicates direct free carrier recombination (ideal case) while n_id_ > 1 suggests that the recombination is trap mediated^35, 36^ with higher values corresponding to the presence of deep traps. In our case, the trap mediated recombination is predominant, as the values are very close to 2, (n_id,spiro-OMeTAD_ = 2.2; n_id,P3HT_ = 2.2; n_id,PCPDTBT_ = 1.7) except in the case of PTB7 (n_id,PTB7_ = 1.4).

### Time resolved photoluminescence decay

Time resolved photoluminescence (TRPL) has been employed to analyze radiative recombination dynamics in perovskite-based solar cells^37–39^. The TRPL response was acquired using time-correlated single-photon counting (TCSPC) technique to measure the resulting decay.

The photoluminescence (PL) spectra of the perovskite\HTM films on the glass substrate (see Figure S4) show a single emission peak, centered at 750 nm, with negligible differences depending on the HTM.

**Figure 5:**
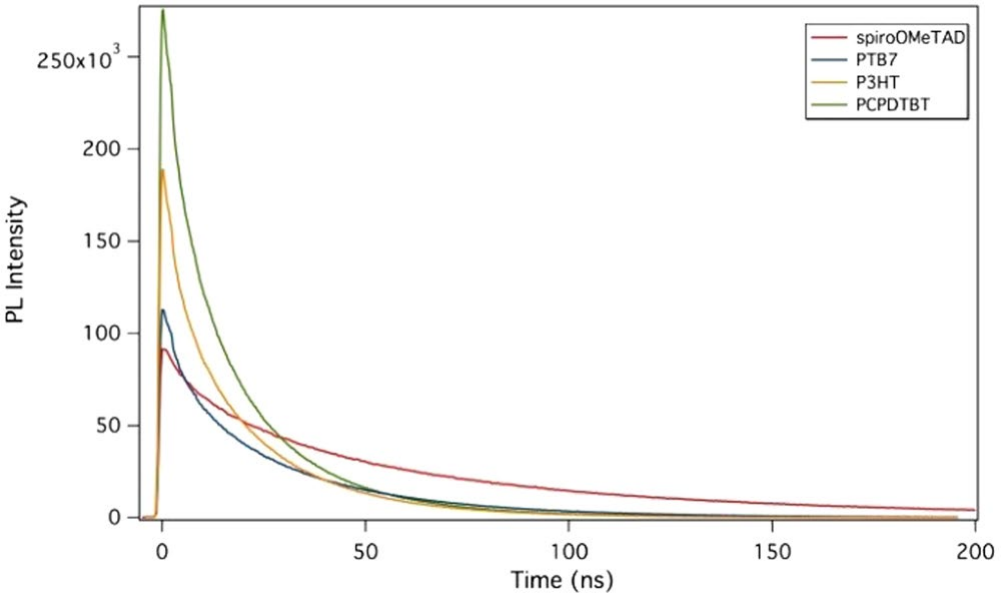
PL decays of the perovskite\HTM films on glass substrate.

The TRPL decays are shown in Fig. 5. These decays can be fitted to a bi-exponential function (Eq. 2)^37, 38^:

2 $$y={y}_{0}+{A}_{1}{{\rm{e}}}^{-({\rm{t}}/{{\rm{\tau }}}_{1})}+{A}_{2}{{\rm{e}}}^{-({\rm{t}}/{{\rm{\tau }}}_{2})}$$

where A_1_ and A_2_ are the relative amplitudes of the components and τ_1_ and τ_2_ the lifetimes associated to the fast and slow recombination, respectively.

The fast one is associated with charge carrier extraction by the layers^37, 39^, which corroborate the efficient charge transfer to the HTM, while the slow component is associated with carriers in the perovskite that do not quench. Both lifetimes change from one HTM to another (see Table 2). Comparing the τ_2_ of the different perovskite/HTM we can conclude that the fastest lifetimes obtained for the polymers which, in turn, imply an increase of recombination within the perovskite, is one of the reasons for the lower efficiency of the respective solar devices.

**Table 2:** TRPL lifetimes obtained fitting the data in Fig. 5 to Eq. 2.

Sample	τ_1_ (ns)	τ_2_ (ns)
MAPI without HTM	—	116.3
MAPI/Spiro-OMeTAD	9.4	60.0
MAPI/PTB7	5.0	30.9
MAPI/P3HT	3.8	21.8
MAPI/PCPDTBT	4.4	20.2

In Fig. 5, the PL decays measured at the same acquisition time (the same excitation time period and recording of their luminescence) for each HTM are shown. As can be seen for PCPDTBT, the HTM that leads to lower device efficiencies, the perovskite luminescence amplitude is higher, which implies that hole transfer (luminescence quenching) from the perovskite to the HTM does not occur efficiently. Thus, the sequence of the efficiency of the hole transfer is PCPDTBT < P3HT < PTB7 < spiro-OMeTAD in agreement with the efficiencies measured and listed in Table 1.

### Photoinduced Transient Techniques

Photoinduced transient techniques have been employed by our group^18, 40, 41^ and others^42, 43^ to study the interfacial carrier recombination dynamics in complete devices, including DSSC, organic and hybrid solar cells and recently PSC^26, 44–46^. As mentioned above, PICE has been demonstrated to be a consistent method to estimate the charge density in different types of solar cells and, together with PI-TPV, allows to evaluate the recombination current (J_rec_).

However, as shown previously^26^, the amount of charge measured using PICE for PSC (in particular MAPI) resulted in an overestimation of the recombination current. To further confirm this fact we have calculated the total amount of charges obtained with PICE and indeed the recombination current does not match the expected value calculated at V_OC_ (see Table ST2). Instead of PICE we employed the PIDC method, which is another technique that has been used to measure capacitance, and thus charge density, in both DSSC and OSC. The differential capacitance can be measured by combining PI-TPV and photoinduced transient photocurrent (PI-TPC) results to find C = dQ/dt/(dV/dt). In contrast with DSSC and OSC, for MAPI solar cells the PIDC and PICE methods does not yield to the same amount of stored charge. We will show later that, for the devices object of this study, the charge density obtained with the PIDC method allows to match (or fairly reproduce) the J_rec_.

**Figure 6:**
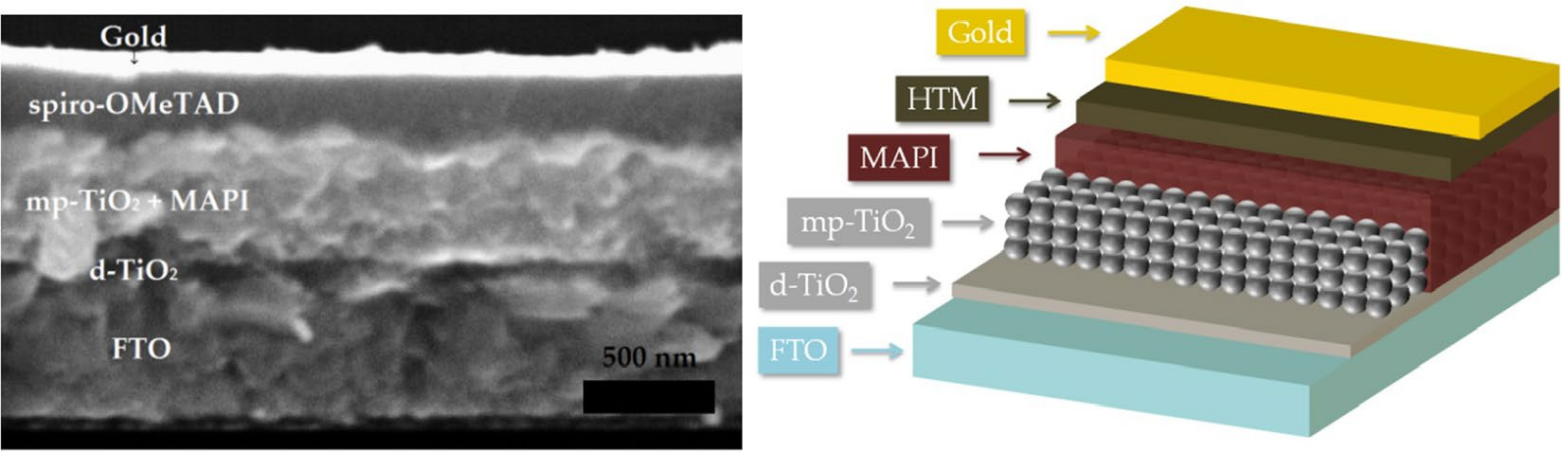
SEM cross section of a MAPI solar cell with spiro-OMeTAD as HTM (left). The scheme with the different layers present in the devices (right).

The quantity dV/dt, which is actually the amplitude of the pulse, can be estimated from PIT-PV at different open-circuit voltages induced by different light intensities. dV/dt increases as the light intensity (thus the V_OC_ decreases). PI-TPV is a quantitative measurement, which allows the study of recombination processes in the device under working conditions. A continuous light bias is applied to the device and a laser pulse generates an additional amount of charges, which cannot be extracted as the device is in open circuit so they have to recombine. All the PI-TPV decays in our efficient solar cells show an exponential decay (see Figure S8) in which the time constant is associated with the interfacial carrier recombination between the electrons in the perovskite and the holes in the HTM, as demonstrated by Montcada *et al*.^44^. This is supported by a very good coverage of the TiO_2_^47^ by the perovskite as can be seen in the cross-section SEM image illustrated in Fig. 6. The perovskite coverage avoids direct recombination channels between the TiO_2_ and the HTM. An irregular and insufficient perovskite coverage of the TiO_2_ leads to bi-exponential PI-TPV decays.

As already observed in many type of solar cells, the charge carrier lifetime, obtained from a single PI-TPV decay, decreases with the increment of photo-induced V_OC_ following the Eq. 3:

3 $$\tau ={\tau }_{0}{e}^{-\beta {V}_{oc}}$$

where τ is the carrier lifetime and β the constant decay.

**Figure 7:**
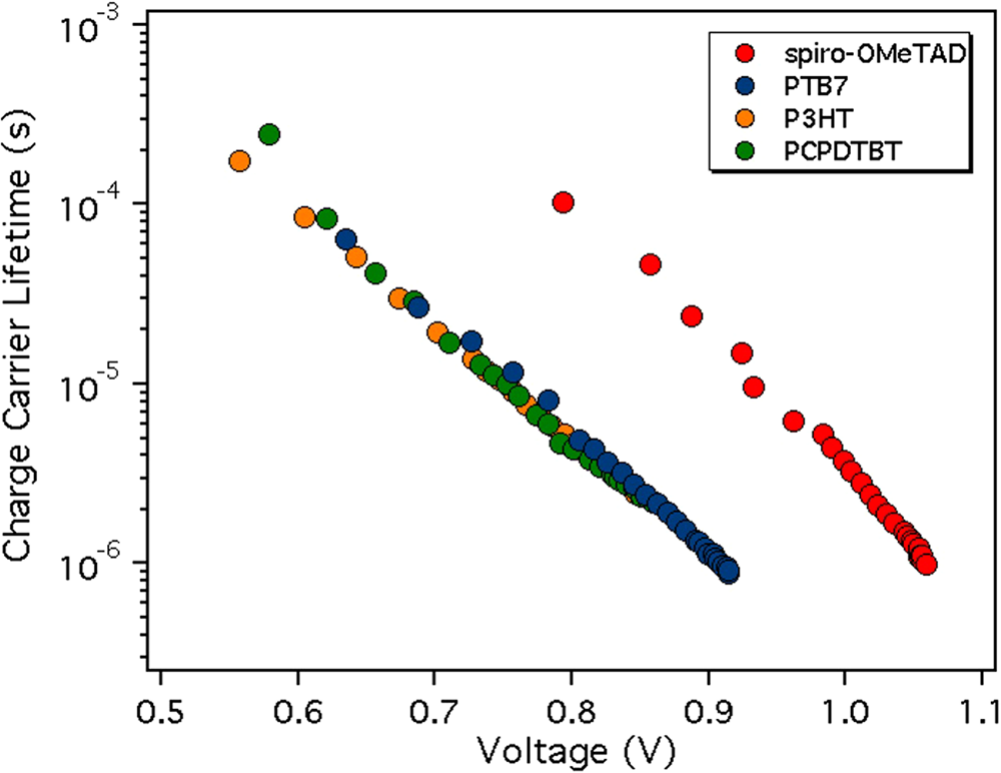
The different τ measured using PI-TPV vs light bias (photoinduced voltage).

All the τs have been estimated from the PI-TPV decays and the result is plotted in Fig. 7. PTB7 and spiro-OMeTAD present the fastest charge carrier lifetimes at 1 sun LI (1 μs) while for P3HT and PCPDTBT the τ is more than 2 μs (values reported Table 3). The calculated βs are 11.5 V^−1^ for PCPDTBT, 14.3 V^−1^ for P3HT, 15.2 V^−1^ for PTB7 and 21.5 V^−1^ for spiro-OMeTAD. Interestingly, the increase of the β value is coherent with the increase of the J_SC_ extracted from the J-V curves.

**Table 3:** Summary of charge density, carrier lifetime, and recombination current for each HTM based PSC.

HTM	Q (nC/cm^2^)	τ (μs)	OF	J_rec_ (mA/cm^2^)	J_SC_ (mA/cm^2^)
Spiro-OMeTAD	157	0.98	6.4	25.0	24.3
PTB7	115	0.92	4.7	26.6	19.6
P3HT	170	2.40	3.8	18.6	18.8
PCPDTBT	85	2.20	3.3	11.7	14.6

Specifically, Q is calculated with the PIDC method, τ is measured with PI-TPV at 1 sun background LI, J_rec_ is calculated from Eq. 4 and J_SC_ is the experimental value obtained from J-V graphs in reverse sweep.

The PI-TPC has been employed in order to find dQ/dt, which represent the amount of charge generated during the pulse (small perturbation). This charge does not change with background light intensity (and thus V_OC_) as can be seen in Figure S9.

**Figure 8:**
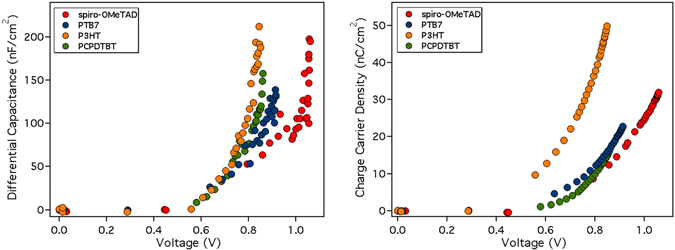
(Left) Capacitance at different voltages (light bias) after subtracting the geometrical capacitance and (right) the charge obtained upon integration of the capacitance at the same voltages.

Figure 8 shows the differential capacitance at V_OC_ measured at different light bias and the charge stored in the device obtained by integrating this capacitance over the voltage. These measurements show that there is a shift in voltage between the spiro-OMeTAD and the polymers in the obtained capacitance in good agreement with the difference in V_OC_ between the solar cells.

**Figure 9:**
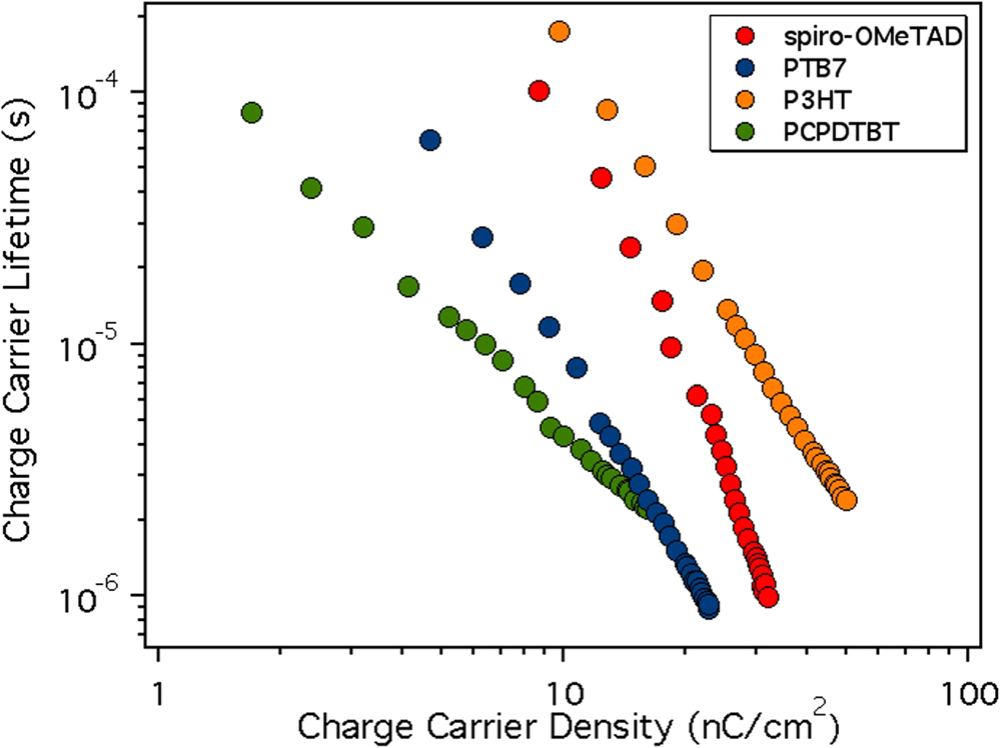
The carriers’ lifetime measured using PI-TPV versus the charge density.

To further evaluate the consistency of the measured charge using the PIDC method, we have related these values with the photocurrent of each device. To do so, we have calculated the recombination current (J_rec_) at V_OC_ in the device under 1 Sun illumination conditions by combining the carrier lifetimes obtained from PI-TPV (Fig. 7) and the charge density. Under these conditions (at open-circuit) the net flux of charge is zero meaning that all the generated charges in the device have recombined. Assuming that the generated current is approximately independent of cell voltage (which could implicitly lead to some degree of error in the current density evaluation) it can be set equal to the J_SC_. Thus, at open-circuit, J_rec_ should be equal to J_SC_. The recombination current J_rec_ is calculated using the following equation:

4 $${J}_{rec}=\frac{Q\,({V}_{oc})}{(1+\frac{\beta }{\gamma })\tau ({V}_{oc})}$$

where Q(V_OC_) is the amount of charges calculated at 1 sun with PIDC, without subtracting the geometric capacitance of the cell (see Figure S10). The τ(V_OC_) is the carriers lifetime obtained from PI-TPV. The factor (1 + β/γ) is also known as the order factor (OF)^26^ in which β is the constant decay from Eq. 3 and γ indicates the slope of log(Q) vs voltage from PIDC (Fig. 8, right). In Fig. 9 we show the direct relationship between charge density and carrier lifetime.

From these calculations, summarized in Table 3, we obtained very reasonable results that approximate the experimental J_SC_, especially for spiro-OMeTAD and P3HT while there is a substantial mismatch in the cases of PTB7 and PCPDTBT. These results confirm that PI-TPV decays actually describe the recombination at the perovskite/HTM interface and, furthermore, that the PIDC is a reliable method to estimate the charge stored in the device.

**Figure 10:**
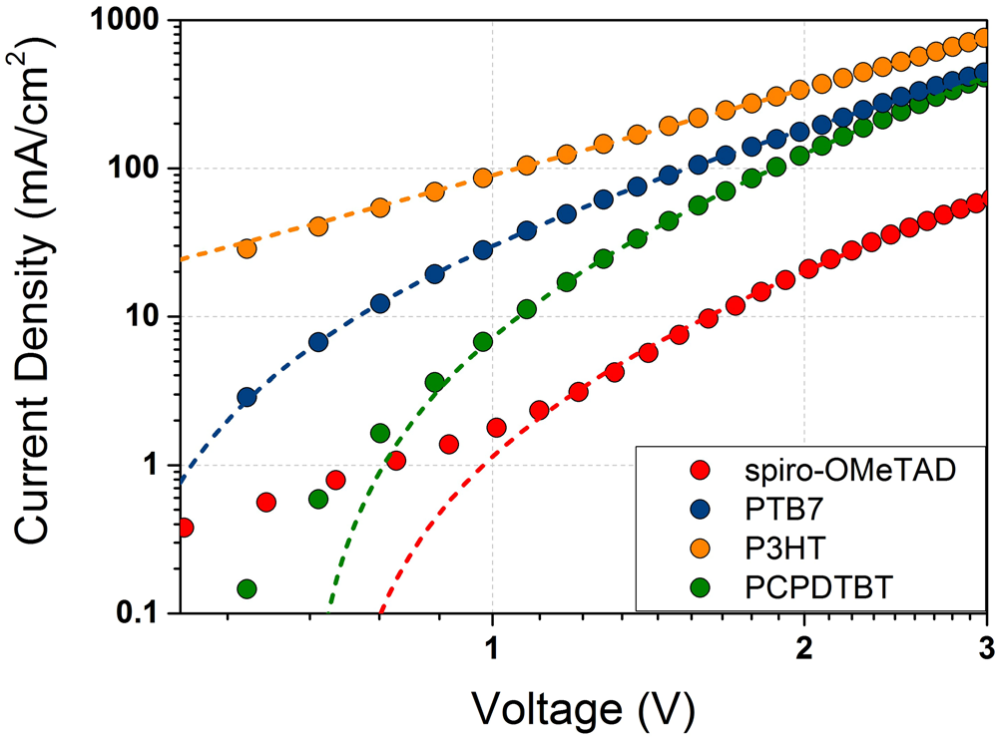
Current density vs. voltage measurements of the devices ITO/PEDOT:PSS/HTM/Au for each HTM used in this study. The dashed lines correspond to the fitting of the experimental data to Eq. 5.

The hole mobility values of the HTMs, as used in the MAPI solar cells, have been measured as described in the methods section. Figure 10 illustrates the J-V graphs of the devices based on the different materials. The obtained values are in accordance with the mobility values reported in the literature^48–50^, confirming that doped spiro-OMeTAD has a better hole transport with a μ_h_ of 1.5 × 10^−3^ cm^2^ V^−1^ s^−1^ while for the rest of the HTMs μ_h_ ranges from 2 to 4 × 10^−4^ cm^2^ V^−1^ s^−1^.

## Discussions

We have fabricated efficient perovskite solar cells using different organic polymer HTMs which have similar HOMO energy levels and we have compared their performance with the standard HTM spiro-OMeTAD. The results show that there is no direct correlation between the HTM HOMO level and the solar cell V_OC_. A first approximation to analyze the origin of the differences observed in V_OC_ leads us to suggest that, on the one hand, in all cases the V_OC_ is limited by trap mediated charge recombination. On the other hand, the measurement of the perovskite luminescence in the presence of the HTM shows that the most efficient hole transfer process occurs in the spiro-OMeTAD sample. In contrast, with PCPDTBT the hole transfer from the perovskite to the HTM is the less efficient of the polymers analyzed in this study. In fact, there is a direct relationship between the photoluminescence quenching and the efficiency of the perovskite solar cells.

The measurement of the functional devices under working conditions shows that there are differences on the obtained capacitance when using PIDC with a clear shift in voltage between the spiro-OMeTAD and the polymers. Moreover, the analysis of carriers’ lifetime shows that the τs are similar under 1 sun irradiation conditions. Furthermore, using the measured charge and the carrier lifetime it was feasible to obtain the recombination current at V_OC_, which is in good agreement with the experimentally measured J_SC_, as demonstrated before. Thus, it is unlikely that for the measured devices the carrier recombination is influenced by existing electric fields and carrier recombination between the electrons in the perovskite and the holes in the HTMs is the main process limiting the solar cell efficiencies.

In addition, the mobility values for the materials used in the solar cells were measured. Indeed, the doped spiro-OMeTAD shows the best charge mobility allowing for devices with thicker hole selective layer and, thus, avoiding potential shorts due to pin-holes.

Finally, we would like to stress that in the case of the spiro-OMeTAD, the LUMO energy gap (the energy difference between the perovskite conduction band and the LUMO of the HTM) was also the biggest in comparison with the polymers. It may well be that this is one of the factors, together with excellent hole mobility and good solubility, that leads to the best solar–to-energy conversion values in perovskite solar cell. Further efforts on the synthesis of new HTM in this direction are being carried out.

## Methods

### Materials

All materials were purchased from Sigma-Aldrich unless differently specified. Methylamonium iodide (CH_3_NH_3_I, MAI) was synthesized by dropping hydroiodic acid (HI, 57% conc. in H_2_O) into methylamine (CH_3_NH_2_, 40% conc. in EtOH, TCI) maintaining the temperature at 0 °C. Then, the mixture was left stirring for 2 hours at room temperature. The solution was rotavaporated at 60 °C until a white precipitate appeared and no solvent remained in the flask. This precipitate was filtered with vacuum, dissolved in methanol, re-precipitated with diethyl ether and filtered again. Finally, it was dried in a vacuum oven at 60 °C overnight. PbI_2_, spiro-OMeTAD, P3HT, PTB7 and PCPDTBT were used as received.

### Device fabrication

FTO coated glasses (R = 8 Ω per square) were etched to obtain the desired pattern using zinc powder and hydrochloric acid (HCl, 2 M). The substrates were properly cleaned by ultrasonication in water with Hellmanex™ detergent, then in de-ionized water and finally in ethanol. A dense layer of TiO_2_ was deposited employing a solution of 0.65 mL of titanium (IV) isopropoxide and 0.38 mL of acetylacetone in 5 mL of ethanol that was spin coated at 3000 rpm for 60 s. Afterwards, the substrates were sintered at 500 °C for 30 min. Then, they were immersed in titanium (IV) chloride (TiCl_4_, 40 mM) at 70 °C for 30 min. They were sintered again at 500 °C for 30 min. Afterwards, mesoporous TiO_2_ was deposited by spin coating a commercial TiO_2_ paste (30 NR-D, Dyesol) diluted in ethanol 1:5.5 (w:w) at 5000 rpm for 30 s. The films were dried at 80 °C just after the deposition and finally sintered at 500 °C for 30 min. The perovskite was obtained with a two-step deposition method, analogue to the one described by Wang *et al*.^51^. In the first step, 1 M solution of lead iodide (PbI_2_, 99%) dissolved in a mixture of dimethylformamide:dimethyl sulfoxide (DMF:DMSO, 92:8) was spin coated at 2000 rpm for 90 s. In the second, a solution of MAI 50 mg/mL in isopropanol:ethanol (75:25), was dripped over the substrate 30 s before the spinning process ends. Successively, the films were annealed for 10 minutes at 100 °C. The HTM deposition differs between the spiro-OMeTAD deposition and the low band gap polymers. Spiro-OMeTAD (1-Material) solutions had a concentration of 72.3 mg/mL in chlorobenzene with the addition of 38.7 μL of tert-butylpyridine and 23.7 μL of Li-TFSI (stock solution of 520 mg/mL in acetonitrile) and it was spin coated at 2000 rpm for 60 s (film thickness around 150 nm). PTB7 (1-Material) and P3HT (Rieke Metals Inc.) had a concentration of 20 mg/mL and PCPDTBT (1-Material) 30 mg/mL in chlorobenzene and the use of any dopant was avoided. Low band-gap polymers solutions were spin coated at 1500 rpm for 30 s. The respective thicknesses, chosen after optimization of each HTM-based cell, are 90 nm for PTB7, 110 nm for P3HT and PCPDTBT. Finally, an 80 nm layer of gold (Kurt J. Lesker Company) was deposited by thermal evaporation.

### Device characterization

#### J-V measurement curves

The J-V curves were measured using a solar simulator (ABET 11000) and a source meter (Keithley 2400). The curves were registered under 1 Sun conditions (100 mW/cm^2^, AM 1.5G) calibrated with a Si-reference cell. The active area of the devices was 0.09 cm^2^.

#### Time-correlated single photon counting

Both TCSPC and PL emission spectra were measured with a Picosecond Fluorescence Lifetime Spectrometer (LifeSpec II, Edinburgh Instruments) equipped with an adequate photomultiplier tube (PMT) and a 470 nm picosecond pulse laser.

#### Photoinduced Time Resolved Techniques

All these measurements were carried out using a homemade system. This setup is based on a ring of LEDs connected to a power supply to allow measurements with different light intensities in order to control the applied bias. For PICE measurements, the solar cell is connected to a homemade electronic board that switches between open-circuit and short-circuit. Both PI-TPV and PI-TPC need a small perturbation in voltage originated by a laser pulse (PTI 3300 Nitrogen laser with dye unit GL-301). All the signals are collected by a Yokogawa DLM2400 oscilloscope and processed with a Labview based homemade software.

#### Mobility measurements

The hole mobility of the HTMs under study has been estimated by means of hole-only devices, fabricated using a diode configuration of ITO/PEDOT:PSS/HTM/Au and by taking current-voltage measurements up to 4 or 5 V. The electric field dependent space-charge limited current (SCLC) mobility was estimated using the following equation:

5 $$J=\frac{9}{8}{\varepsilon }_{r}{\varepsilon }_{0}{\mu }_{h}\frac{{V}^{2}}{{L}^{3}}{e}^{0.89b\frac{\sqrt{V}}{\sqrt{L}}}$$

where J is the current density, L is the film thickness of active layer (around 100 nm), μ_h_ is the hole mobility, *ε*_r_ is the relative permittivity of the material (we assumed a value of 3, a fair assumption for organic materials), *ε*_0_ is the permittivity of free space (8.85 × 10^−12^ F/m), b is the field activation factor and V is the effective voltage in the device (which is the applied voltage corrected for the built-in, V_bi_, and the voltage drop due to series and contacts resistances of the device).

#### Scanning electron microscopy (SEM)

The SEM images were acquired to estimate how the different layers of the devices were constructed. A Jeol JSM-6400 microscope was used to obtain the images.

## Electronic supplementary material

Supplementary Information
