# Supplementary material for: Charge Injection, Carriers Recombination and HOMO Energy Level Relationship in Perovskite Solar Cells
Source: Sci Rep. 2017 Jul 21;7:6101. doi: 10.1038/s41598-017-06245-5 (PMC5522453; doi:10.1038/s41598-017-06245-5)
Supplement: Supplementary file 1 — Supplementary Information [file 41598_2017_6245_MOESM1_ESM.pdf]

Charge Injection, Carriers Recombination and HOMO Energy Level Relationship in Perovskite Solar Cells.

(Supporting Information)

Jesús Jiménez-López[1], Werther Cambarau[1], Lydia Cabau[1], Emilio Palomares[*,1,2]

[1]Institute of Chemical Research of Catalonia (ICIQ), Barcelona Institute of Science and Technology, Avda. Països Catalans, 16, E-43007, Tarragona, Spain

[2]ICREA, Passeig Lluis Companys, 28, E-08018, Barcelona, Spain

*epalomares@iciq.es

Device Characterization

J-V curves

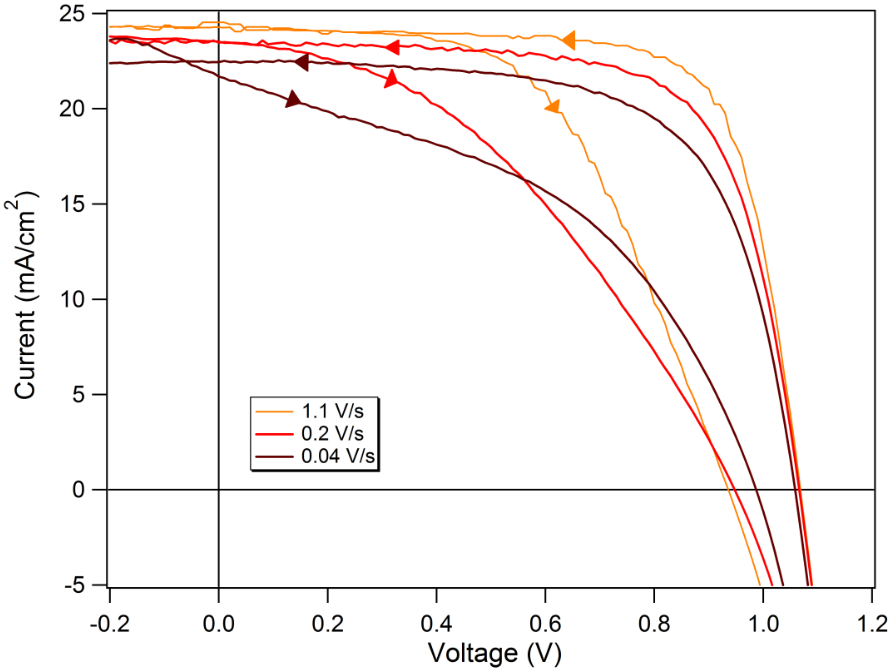

Figure S1. Forward and reverse scan J-V characteristics acquired at different scan rates for the spiro-OMeTAD based solar cells.

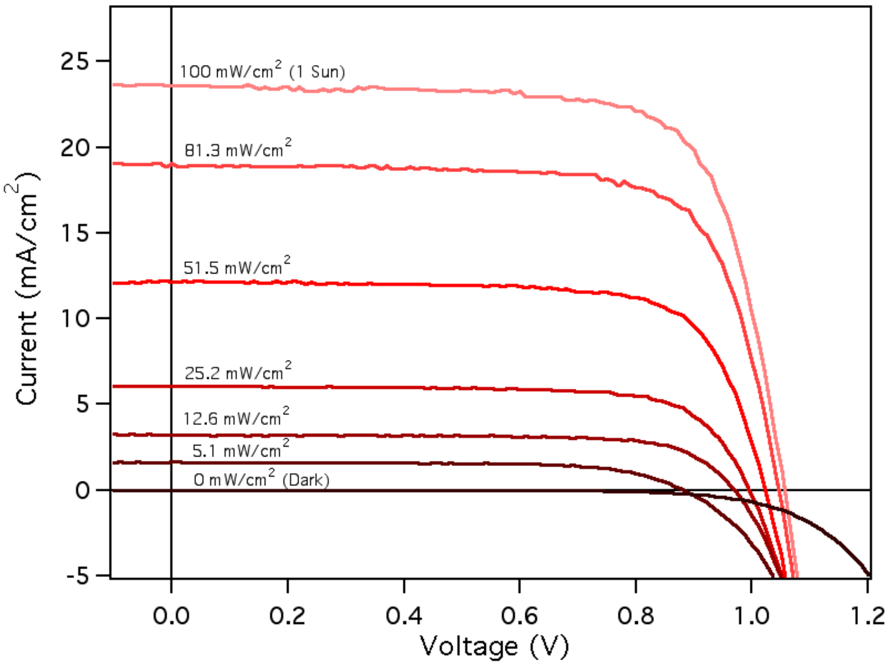

Figure S2. J-V characteristics measured in reverse scan at different light intensities (using appropriate neutral density filters) for the device FTO/TiO_2/MAPI/spiro-OMeTAD/Au.

V_{OC} vs time

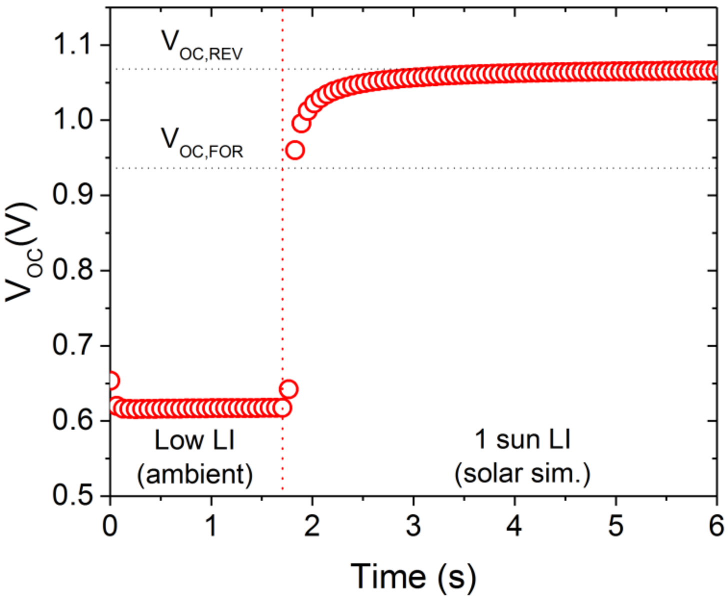

Figure S3. Stabilization time of V_{OC} for the spiro-OMeTAD based cell (see Table 1 of the manuscript). The cell is kept at low LI (ambient light) until the solar simulator is switched on and a 1 sun LI is applied.

Photoluminescence

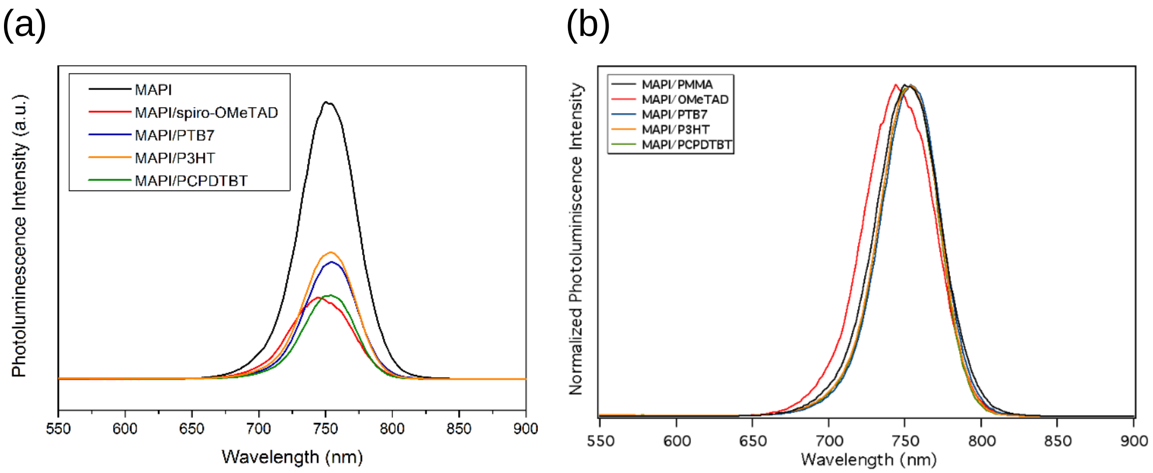

Figure S4. Photoluminescence spectra of the different films prepared with the HTM on top of the perovskite: (a) absolute values; (b) normalized values. Analyzing the decay focus on the emission peak, the TCSPC was calculated.

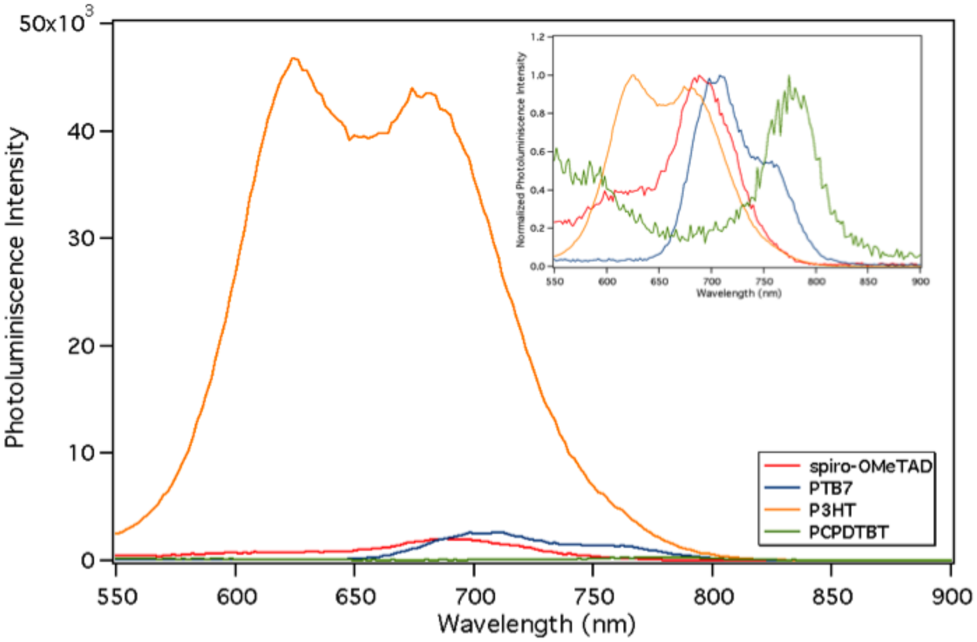

Figure S5. Photoluminescence spectra of the different HTM employed in this study. The inset shows the normalized spectra.

Photoinduced transient measurements

HTM	Sweep	V_{OC} (mV)	J_{SC} (mA/cm^2)	FF (%)	PCE (%)
**Spiro-OMeTAD**	forward	936	24.4	55.1	12.6
	reverse	1068	24.3	73.6	19.1
**PTB7**	forward	904	19.8	44.0	7.9
	reverse	947	19.6	63.2	11.7
**P3HT**	forward	797	16.3	33.3	4.3
	reverse	877	18.8	65.9	10.9
**PCPDTBT**	forward	853	13.4	42.8	4.9
	reverse	911	14.6	56.0	7.4

Table ST1. Photovoltaic parameters of the devices studied with photoinduced time resolved techniques.

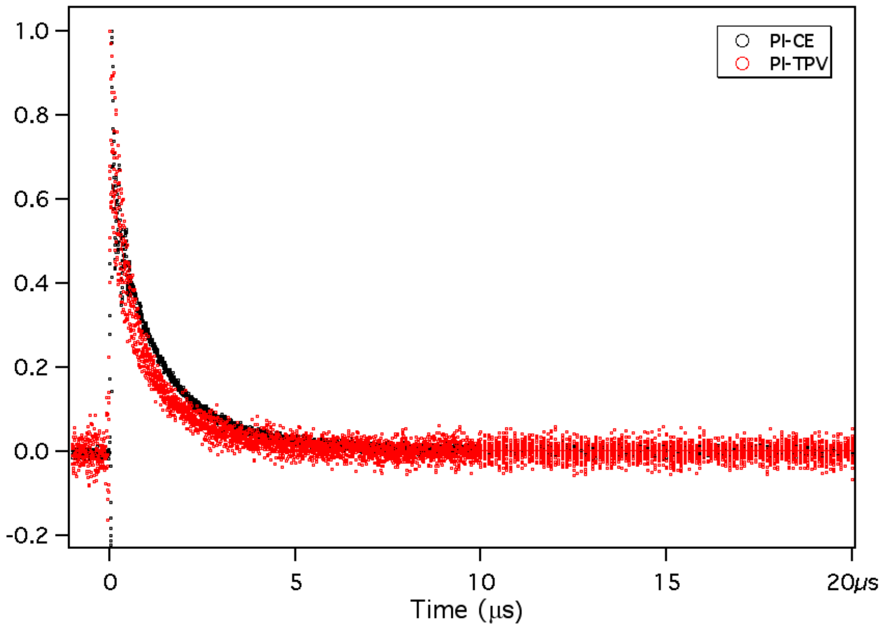

Figure S6. Normalized decays of PICE vs PI-TPV at 1 sun LI background.

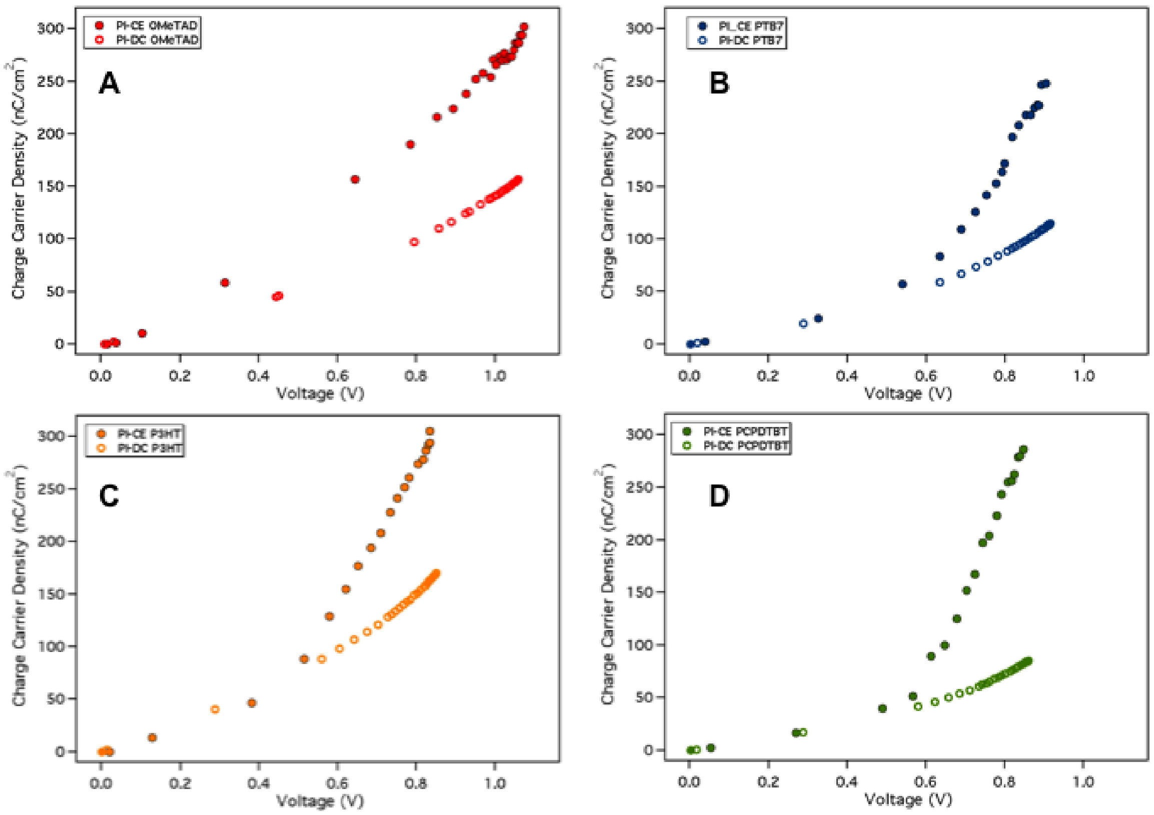

Figure S7. Comparison between all the different HTM based cells in terms of extracted charge obtained with PICE (solid symbols) or PIDC (void symbols).

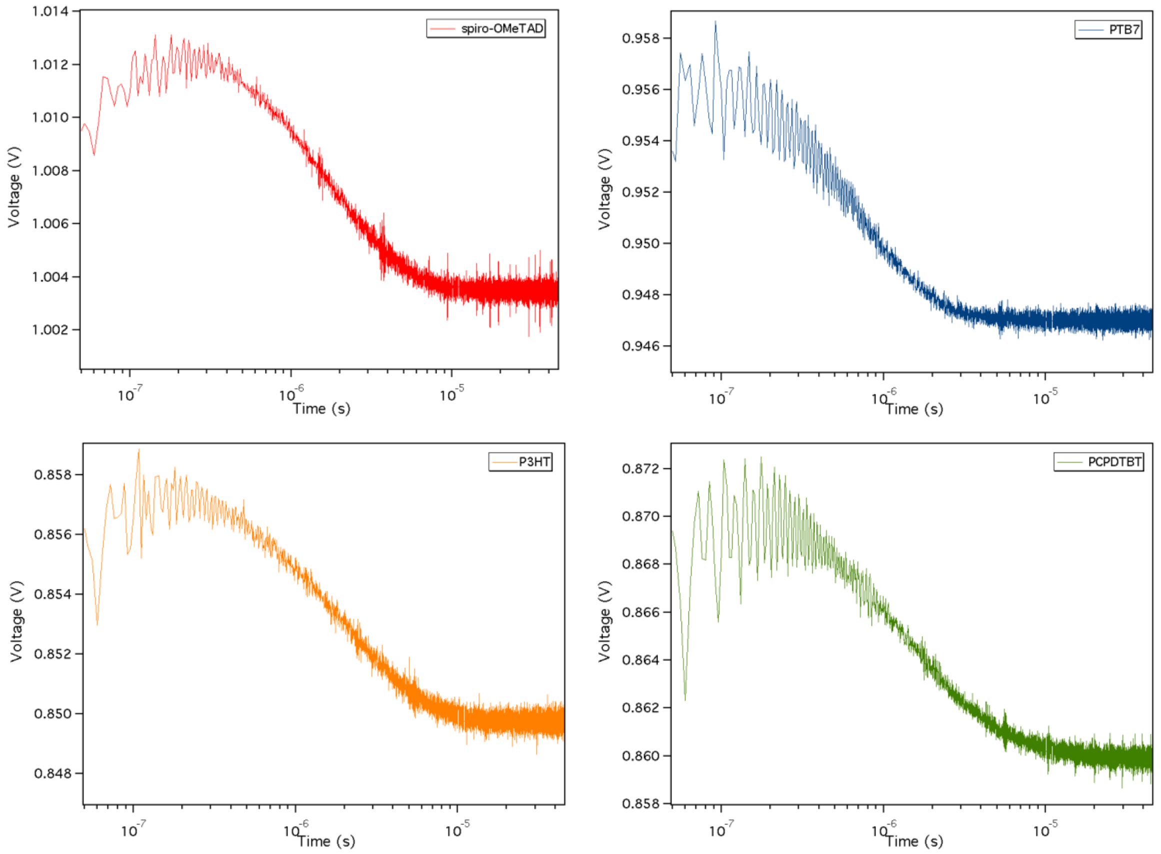

Figure S8. PI-TPV decays for each HTM based cell at 1 sun LI background.

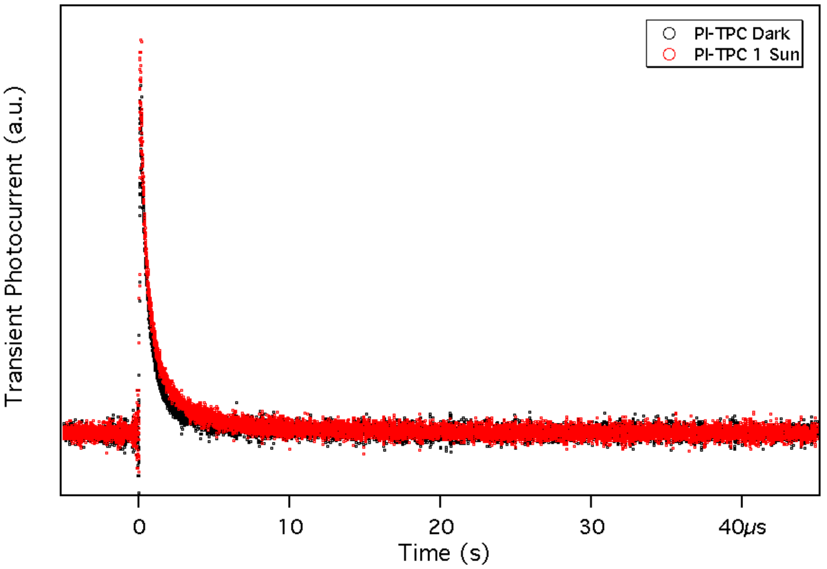

Figure S9. PI-TPC decays at 1 sun and dark background LI for a spiro-OMeTAD based cell. The values are corrected for the respective baselines and are not normalized.

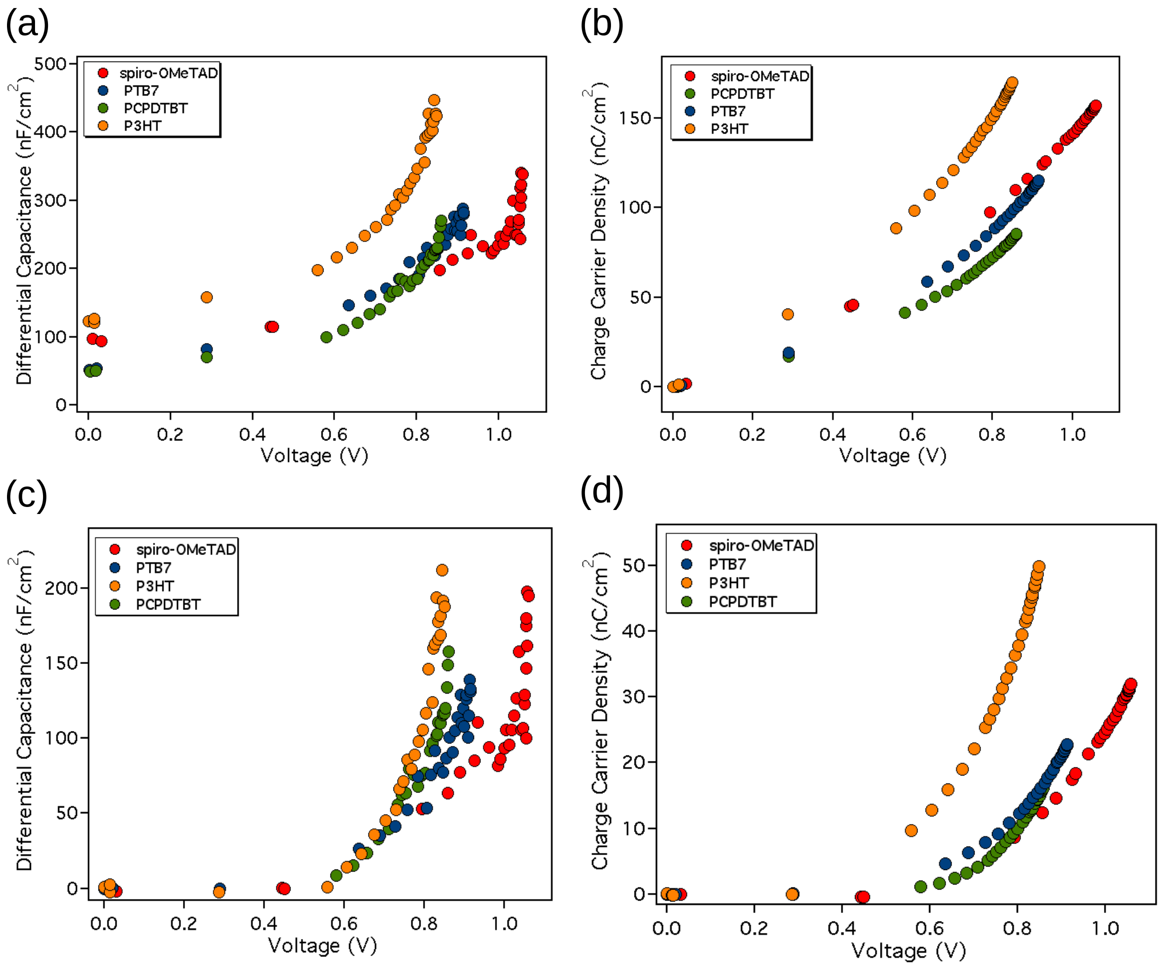

Figure S10. Differential capacitance (a, c) and charge density (b, d) versus voltage obtained with PIDC without subtracting the geometric capacitance (a, b) and subtracting the geometric capacitance (c, d).

HTM	Charge meas. type	Q (nC/cm^2)	τ $^{\text{PI-TPV}}$ (μs)	OF	J_{rec} (mA/cm^2)	J_{SC} (mA/cm^2)
**Spiro-OMeTAD**	PICE	302	0.98	9.0	34.4	24.3
	PIDC	157		6.4	25.0	
**PTB7**	PICE	248	0.92	5.2	51.4	19.6
	PIDC	115		4.7	26.6	
**P3HT**	PICE	329	2.40	6.3	21.9	18.8
	PIDC	170		3.8	18.6	
**PCPDTBT**	PICE	286	2.20	3.4	37.7	14.6
	PIDC	85		3.3	11.7	

Table ST2. Summary of charge density, PI-TPV lifetime at 1 sun LI and calculated recombination current at V_{OC} according to Equation 5 of the manuscript. The J_{SC} is the experimental value obtained from J-V graphs in reverse sweep.
